# Supporting Personal Growth in Childhood, Adolescent and Young-Adult Cancer Survivors Through Challenges in Nature — A Qualitative Study of WAYA Wilderness Programme Participation

**DOI:** 10.1177/27536130241238150

**Published:** 2024-03-08

**Authors:** Mats Jong, Trine Stub, Miek C Jong

**Affiliations:** 1 Department of Health Sciences, 6311 Mid Sweden University, Sundsvall, Sweden; 2 National Research Center in Complementary and Alternative Medicine (NAFKAM), Department of Community Medicine, Faculty of Health Sciences, 8016 The Arctic University of Norway, UiT, Tromsø, Norway

**Keywords:** adolescent, adventure therapy, childhood cancer survivor, ecosophy, ecotherapy, forest bathing, mindfulness, outdoor-based therapy

## Abstract

**Background:**

Childhood, adolescent and young-adult (AYA) cancer survivors often experience health problems due to late or long-term effects of their cancer or the treatment thereof. The general population gains health benefits from immersion in nature, and nature-based programmes seem to be an intervention that can promote health among childhood and AYA cancer survivors.

**Objective:**

To explore the impact of the WAYA wilderness programme on the health of childhood and AYA cancer survivors.

**Methods:**

The study had a qualitative approach, with data from individual interviews (n = 18) 3 months after completion of the WAYA programme. In addition, case report data was collected during follow-up talks (1, 2 and 12 months after the programme) (n = 19). The WAYA programme consisted of an 8-day expedition, followed 3 months later by a 4-day base camp. The programme included activities such as hiking, backpacking, kayaking, rock climbing, bushcraft and mindfulness. Data was analysed according to a qualitative content analysis. The consolidated criteria for reporting qualitative research (COREQ) were followed.

**Results:**

An overarching theme was identified: *“Personal growth from challenges in nature supported by deep connections with others”*. In 4 additional themes, participants’ experiences describe how deep personal connections arose, as they developed a feeling of being able and competent in nature. Nature provided a space that supported relaxation and respite from everyday challenges and stimuli, which also led to an experience of being more connected to nature.

**Conclusion:**

The WAYA programme was experienced as being of support to childhood and AYA cancer survivors. The programme provided them with skills and tools to be safe in nature. When connected to nature, the participants developed trust and self-confidence, personal growth, relaxation and recovery from stress. Their engagement in outdoor activities continued after completion of the programme, when they returned to everyday life at home.

## Introduction

During the last 50 years, cancer survival rates in children, adolescents and young adults (AYAs) exceeded 80%.^1-5^ AYAs, defined by the National Cancer Institute as cancer survivors between 15 and 39 years of age,^6^ suffer to a great extent from late effects of their cancer or treatment. For example, they have a higher risk of mortality from remission or secondary cancer types, a higher risk of severe disabling chronic health conditions,^3,7^ suffer more psychological distress, and have a lower quality of life than the general population.^1,8^ Post-cancer fatigue appears to be a common phenomenon that affects up to 61.7% of cancer survivors.^9^ Psychological distress among AYA cancer survivors is dependent on multiple complex issues as a consequence of a cancer diagnosis, being treated, and current stage in life, and is, for example, related to fertility, education, early-career goals, social interaction, family functioning and financial status.^10-12^ Compared to older adults, AYAs face unique social and emotional challenges related to their appearance (eg, hair loss, weight gain), job loss, fertility and financial stress.^13^ They often have to deal with many changes in their lives, such as school, work and thinking about starting a family someday.^14^ Also, during their teenage years, they might struggle with figuring out who they are, dealing with their changing bodies, and wanting more independence from their parents.^14^ One less-studied aspect is how AYA cancer survivors’ experience of loneliness affects their psychological well-being. Ernst et al.^15^ studied a registry-based sample of n = 633 adult long-term childhood cancer survivors and found that loneliness was reported by 18%, and that it was a risk factor for persistent anxiety symptoms and suicidal ideation. Having difficulties in interacting with others was brought up by Lehman et al.^16^ as an additional negative consequence of a cancer experience. AYA cancer survivors also report that the cancer experience can have positive consequences, with a more positive view of self and life, compassion for others and close relationships. Similarly, personal growth and accepting cancer as a part of one’s life were reported by a sample of adult long-term cancer survivors by Foley et al.^17^ In an extensive survey of the health profiles of long-term cancer survivors by Schultz et al,^18^ younger survivors to a higher degree reported that their health was negatively affected.

As seen in the systematic review by Mygind et al,^19^ immersion in nature supports health and well-being and is increasingly used in public health interventions. The review finds that both passive and active activities in nature may support different aspects of health, no matter whether the target group is healthy, vulnerable or sick. However, the general quality of the articles that were investigated was low, and therefore no general conclusions regarding their evidence base could be drawn. It is suggested in this review that future research needs to consider the complexity of pathways between immersive nature experiences and health outcomes. Further, that research needs to be guided by logical theoretical models that can explain possible changes. Similar conclusions were made by the present research team after performing a scoping review of a similar topic area, but with a focus on childhood cancer survivors — the role of nature in different interventions was poorly described.^20^

A theoretical model to explain how nature immersion supports the health and well-being of those exposed to it is suggested in a review by Kuo.^21^ In this review, 21 different pathways were identified that offer possible explanations, and these are related to environmental factors, physiological and psychological states, and behaviours or conditions. In particular, improved immune function is identified as a possible explanatory factor. The line of reasoning is that while spending time in nature, humans are exposed to active stimuli (eg, phytoncides, negative air ions, mycobacteria, biodiversity, natural sounds), that alter physiological and psychological states (relaxation, awe, vitality, adiponectin), and behaviour (physical activity, sleep, social ties), leading to improved health outcomes.^21^ With physical activity as an example of a behavioural factor that is interrelated with exposure to nature, the link appears sound, since spending time in nature has been reported to increase physical activity.^22^ A previous study of young adult cancer survivors found that a week out in nature increased their physical activity for up to 3 months after the programme, compared to a waitlist control.^23^ In general, there is evidence-based consensus that physical activity positively influences most aspects of health among the general population.^24^ Similarly, exercise and physical activity are considered to be safe and to generally have positive health effects among cancer survivors, such as a better quality of life and less cancer-related fatigue.^25^

To account for the complex relationships between immersive nature experiences and health outcomes, it is necessary to understand how these are perceived by cancer survivors. This is the focus of a meta synthesis of published qualitative research by Blaschke,^26^ where part of the material included AYA participants (most participants were 32-80 years of age). The study’s conclusion proposes that exposure to nature provides a “secure base” that offers a safe and trustful context, where it is possible to develop new perspectives and connections to oneself and others. It is further suggested that outdoor-based programmes may contribute to building relationships with peers, and also support positive lifestyle changes.^27^ Nevertheless, Blaschke acknowledges that further research is necessary in order to identify, understand and safely implement outdoor-based programmes as supportive care practice for cancer survivors.^26^ In the development of the Wilderness programme for AYA (WAYA) cancer survivors, Jong et al.^28^ performed a scoping review in order to map the concept, content and outcome of wilderness programmes for childhood and AYA cancer survivors. In the study, it is recommended that further research should describe the direct and indirect role of nature more explicitly, as this information was lacking. In the scoping review, only 1 study with a qualitative design was identified that had a direct focus on the experience of taking part in a wilderness programme.^27^

From reviewing the literature, as shown above, it is more or less generally accepted that immersion in nature and physical activity can support the health of a general population and among cancer survivors. There is, however, almost a total lack of research of how nature-based programmes for AYA cancer survivors affect aspects of health. Further insight is thus needed into how wilderness or nature programmes impact the health and lives of childhood and AYA cancer survivors. AYAs suffer from a wide variety of health problems, and spending time in nature plays a promising role in supporting their health and well-being. Due to the lack of studies and systematically developed knowledge of the topic of the role played by nature-based programmes in supporting health among AYA cancer survivors, the WAYA wilderness programme was developed.

**Figure 1.**
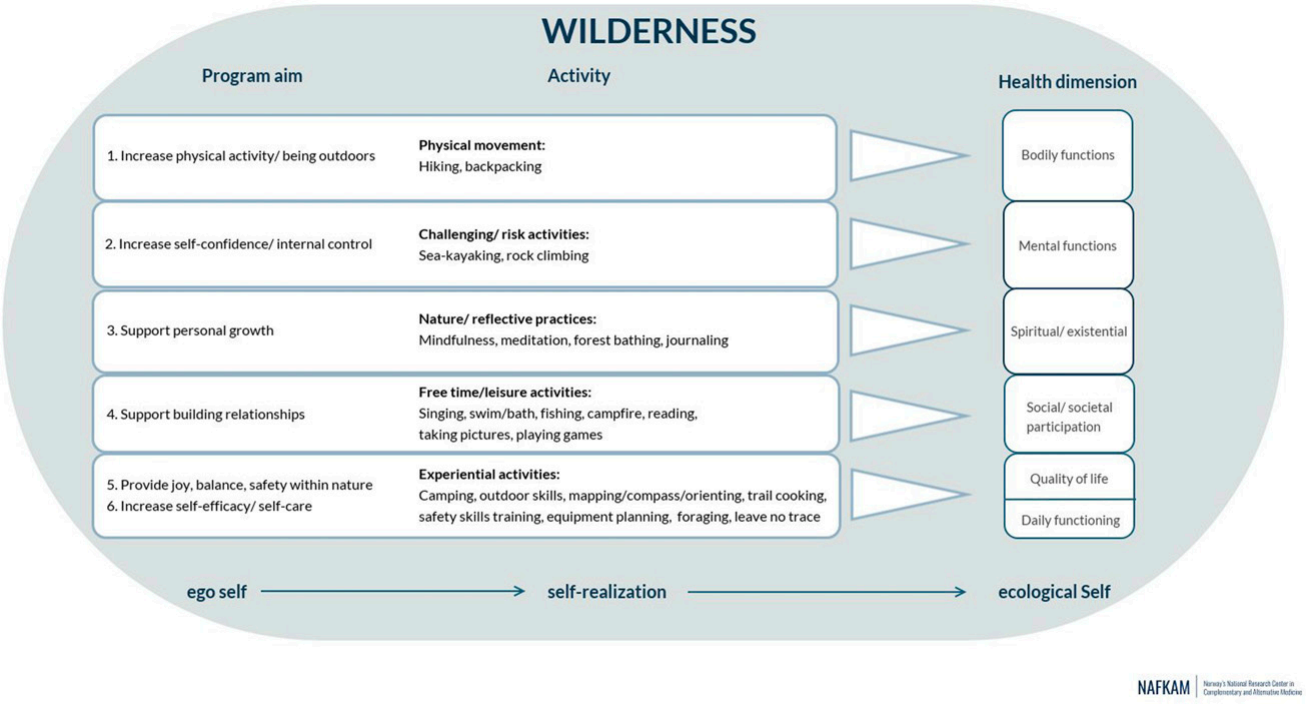
Schematic overview of the WAYA programme. Used with permission due to an open-access Creative Common CC BY licence. Publication Jong et al.^31^

The WAYA programme and the mixed-methods study design were developed and based on the results from the scoping review by Jong et al.^20^ in collaboration with experts in outdoor life and organisations for childhood and AYA cancer survivors in Sweden. The programme consists of an 8-day wilderness expedition, followed 3 months later by a 4-day base camp, with activities such as hiking, backpacking, kayaking, rock climbing, mindfulness and bush crafting. An inherent goal of the programme is to promote health by supporting long-term interest and engagement in outdoor activities, and the development of relationships with others. The programme was pilot tested in a randomised controlled study (RCT) to assess feasibility and safety, where the comparison group attended a holiday programme of 8 + 4 days in a spa hotel.^29^ A detailed description of the programme’s development and content can be found in previously published articles by Jong et al.^30,31^ WAYA is based on the conceptual positive health model, which views health as “the ability to adapt and self-manage in the face of social, physical, and emotional challenges”.^32^ Rather than focusing on treating disease, the programme aims to promote health by supporting increased physical activity, self-esteem, self-efficacy, self-care, personal growth, and supportive relationships among participants. The theoretical framework of the programme is found in the ecosophy theory of Naess.^33^ By participating in WAYA, participants have the opportunity to establish a connection with nature and expand their sense of being, or self-realisation, connected to the ecological Self. This shift is hypothesised to be supportive of an increased sense of well-being, personal growth and meaning making, all of which have been linked to eudaimonic well-being. This, in turn, is thought to be of great importance in dealing with significant life challenges such as surviving cancer.^34^ The WAYA programme was evaluated to be developmentally appropriate and of interest for this population, and just as safe as spending a relaxing holiday at a spa hotel.^29^ An overview of the programme and its activities in relation to the positive health model can be seen in Figure 1, which provides a visual representation of the programme’s theoretical underpinnings.

The objective of this study was to explore the impact of the WAYA wilderness programme on the health of childhood and AYA cancer survivors.

## Materials and Methods

The study utilised an exploratory qualitative approach, which involved collecting data through individual interviews 3 months after the wilderness expedition and from the case report form (CRF) during follow-up talks (1, 2 and 12 months after the 8-day expedition). The reason for using this qualitative approach was to gain insight into the participants’ health, opinions, perspectives and experiences regarding programme participation. Qualitative interviewing operates under the assumption that the interviewee’s perspective is meaningful and valuable and can be made explicit for others to understand.^35^ This methodology is well-suited for exploring participants’ perspectives on wilderness-based programmes for cancer survivors.

All collected data underwent qualitative content analysis, inspired by the methodology described by Graneheim and Lundman.^36,37^ The objective of an analysis is to condense and analyze text originating from written, verbal or visual documentation.^38^ Part of the data collected in this study that concerned evaluation of the content (programme activities) and facilitators of the programme was previously analysed with the objective of investigating the acceptability of the wilderness programme among the participants.

In the preparation and reporting of the study, the consolidated criteria for reporting qualitative research (COREQ) were used^39^ (Suppl l Appendix COREQ).

### Sample and Recruitment

In 2021, a total of 19 people participated in WAYA, divided into 2 groups. An overview of the programme is described briefly above, and in more detail in a previously published article.^31^ The recruitment of participants into WAYA followed a purposive sampling approach directed mainly at those who were: 16-39 years of age, and a member of the Swedish cancer organisations Ung Cancer (Young Cancer) or the Swedish Childhood Cancer Fund. Further inclusion criteria were that eligible participants could have or could have had any type of cancer at any time during their life (from birth up to 39 years of age) and were able to walk at least 2 km without a pause (walking aids or other support allowed). Active cancer treatment or a medical condition that would impede safe travel and participation in the programmewere regarded as an exclusion criterion. Articles with study and contact information were posted on the social media platforms and newsletters of the member organisations. Participants did not receive specific information about the objectives of the wilderness programme, but were informed about the general aim of the feasibility study.^30^

### Data Collection

#### Interviews

About 1 week after participation in the 4-day WAYA basecamp programme (3 months after the 8-day expedition), individual interviews were conducted with 18 out of 19 participants, either face-to-face in video conferencing (n = 16) or by telephone (n = 2). One participant opted not to be interviewed, due to illness in the family. The interviews were semi-structured and focused on areas of interest, including the general impression and experience of the programme and its activities, perceptions of facilitators and other participants, the nature experience, health aspects, risks, incidents and motive for participation (Suppl 2 Appendix interview guide). The interviewer asked open-ended questions related to the topic areas and followed up with further questions such as “Please elaborate …” or “What did you mean by that …?” when necessary to encourage participants to delve more deeply into the topic. All topics in the interview guide were given similar attention by the interviewer. The interviews lasted between 20-74 minutes, with an average duration of 43 minutes, and were conducted by the second author (TS), who was not involved in the supervision of the WAYA programme. All 18 recorded interviews were transcribed by a professional transcription service and checked for accuracy against the recordings by the 3 authors. The results of the interviews, which relate to the acceptability, feasibility and safety of programme participation, have been previously reported. All interviews were conducted in October 2021.

#### Case Report Forms — Open Text Documentation

Follow-up talks with all 19 participants were held 1, two and 12 months after the expedition by the first author (MJ). During these talks, aspects of lifestyle, participants’ health, physical activity and life events were discussed, as well as reflections on their programme participation experience. The follow-up talks with participants differed from interviews in the sense that they were not structured and were performed by 1 of the facilitators (first author) with the inherent aim of identifying outdoor activity areas in which the participants could continue to develop skills and experience. Accordingly, they were then coached about realistic goals for their own outdoor practice. The content of the follow-up talks was summarised in the CRF. In the follow-up talks 12 months after the expedition, all previously raised topics were reflected on once again, and participants were encouraged to relate to their wilderness experience in a broader perspective, for example in terms of health, physical activity and their relationship with nature. These follow-up talks after 12 months were recorded and subsequently transcribed.

### Data Analysis

In line with the methodology of Graneheim and Lundman,^37^ the texts from the interviews (636 double-spaced A4 pages) and follow-up talks (35 pages) were brought together into 1 text, which constituted the unit of analysis. As a first analysis step, transcripts were read several times and simultaneously, meaning units (sentences, paragraphs) were identified and tagged with temporary codes that represented their manifest content. The 6 overarching aims of WAYA (Figure 1) served as guiding questions to identify meaning units of relevance to the objective.^31^ In the second step, meaning units with similar content were sorted into content areas, based on the guiding questions (often sorted into 2 or more domains, as the units could not be split without losing meaning and context). The 6 overarching aims with underlying content areas (domains) and subsequent underlying categories made up the coding tree (Suppl 3 Appendix Coding Tree). The text content was rich in terms of deeper meaning. The third step took the form of an inductive content analysis where the meaning units of each category were re-read to identify the latent meaning of the text. As themes were identified, the coded meaning units were rearranged, based on their similarities at a higher level of abstraction. The identified themes are connected to the overarching aims of the WAYA programme (Figure 1), but since the “meaning” relates to something deeper about the participant experiences, the “aims” can be present in more than 1 of the themes. In the words of Graneheim & Lundman (2004), “A theme can be seen as an expression of the latent content of the text”.^37^ In the findings, quotes are shown to provide examples of data, and how content has been sorted and interpreted. The abbreviation ‘IP’ denotes Interview Person, to denote representation of different participants. To ensure reliability, the analysis process was an iterative process in which all 3 authors were involved in reading, analysing, sorting, re-reading, re-sorting, discussing and agreeing upon identified themes. In another step to support credibility and confirmability, 3 facilitators of the expeditions in the WAYA programme were asked to read and comment on the identified themes, resulting in minor changes in wording. The concept of “information power” can be used as a model to guide the sample size in qualitative research (Malterud et al.).^40^ When data is collected for a broader objective, more participants (data) are needed, and when data is richer, fewer participants are needed to meet the objective.^40^ The NVivo qualitative software program was used as a tool to assist in data management and analysis.^41^

### Ethics

During recruitment for the study, written informed consent was obtained from all participants. They were informed both verbally and in writing about the study’s aim (investigating the feasibility and acceptability of performing an RCT), and that participation was voluntary. Participants were made aware that all activities within the programme were voluntary and that they could withdraw their consent at any time, without providing a reason. The consent statement also provided details of how the collected data (digital recordings) would be used and stated that any personal information would be kept confidential. Additionally, the Swedish Ethical Review Authority approved the study (Dnr 2020-00239). The protocol for the full WAYA programme was published in 2022,^30^ and the study was pre-registered at https://www.clinicaltrials.gov (NCT04761042).

The second author (TS), a female research professor in health sciences from Norway, conducted interviews with all participants 3 months after the programme expedition. Despite having no prior relationship or contact with the participants, TS has experience from conversing with people with chronic illnesses. This experience allowed TS to support the participants in being fully open about their programme experiences, both positive and negative. The first author (MJ) and last author (MCJ), both academic researchers in the field of health sciences, designed and facilitated the intervention study. MJ, a registered nurse with experience from coaching and motivational interviewing, conducted all coaching talks and 1-year follow-up conversations. Additionally, MJ is an authorised outdoor survival instructor. Throughout the analysis process, all authors continuously discussed the importance of being aware of their own pre-understanding, to present transparent and unbiased results. All 3 authors are experienced in qualitative research methodology. Named that are mentioned in the result section have been changed to preserve confidentiality.

## Results

The participants in the study were 7 men (36.8%), and twelve women (63.2%), and their mean age was 29.4 years (SD ± 3.8). Their age at cancer diagnosis varied between 1.5-29 years (SD ± 8.4). For more demographic characteristics, see Table 1.

**Table 1.** Demographic Characteristics of Study Participants (n = 19).

Demographic Characteristics	n*/%*
Ethnicity	
One or both parents from Sweden	18/94.7
Both parents from outside Sweden	1/5.3
Marital status	
Living with partner/married	9/47.4
Single	10/52.6
Level of education	
Comprehensive	1/5.3
High school	6/31.6
College/university	10/52.6
Other	2/10.5
Occupational status	
Full-time work	9/47.4
Part-time work	4/21.1
Full-time education	2/10.5
Unable to work	4/21.1
Relative economic situation *(how often do you have good finances to be able to do the same things as your friends)*	
Always	9/47.4
Often	8/42.1
Sometimes	1/5.3
Seldom	1/5.3
Primary type of cancer	
Hematological	6/35.3
Brain	6/35.3
Testicular	2/10.5
Thyroid	2/10.5
Osteosarcoma	1/5.3
Breast	1/5.3
Timespan since last treatment	
<3 months	4/21.1
4-11 months	1/5.3
1-5 years	5/26.3
>5 years	8/42.1
Presently under treatment	1/5.3

In the content analysis, 4 themes and ten subthemes were identified. At a higher level of abstraction, the overarching theme of**: Challenges in nature and deep connections with others — a foundation for personal growth** is present in the themes. An overview of the themes and subthemes is shown in Table 2.

**Table 2.** An Overview of Themes and Subthemes.

Themes	The Surrounding Nature and Similar Life Experiences as a Context for Developing Deep Connections with Others	Developing an Experience of Being Able and Competent in Nature	Nature: a Space that Supports Relaxation and Respite from Everyday Challenges and Stimuli	An Experience of Becoming Genuinely Connected with Nature
Subthemes	A sense of finding a new family	An experience of developing trust in one’s own physical and mental capabilities	Peace and calmness as induced by being in nature	Old forests and uninhabited seashores as support for reflection, learning, and being in the moment
	Nature, programme activities and similar life stories as catalysts for deep connection	Growing by challenging one’s own personal boundaries in a safe context	Reconnecting with nature’s calming properties after programme participation	A deliberate choice to be present, while being in nature
	Finding personal support in the shared stories	A growing interest in wanting to be active in nature		

### THEME: Surrounding Nature and Similar Life Experiences as a Context for Developing Deep Connections with Others

An inherent objective within the wilderness programme was to support personal relationships between all participants, and this was also a recurring element of participants’ stories during the follow-up interviews and talks. Deep and close relationships were quickly formed due to guided exercises (reflective talks and mindfulness meditations) and outdoor activities, where it was easy to spontaneously offer help to others or ask for support. The constant immersion in the different and changing nature elements and the similarities in life experiences provided a natural base for interaction between participants.

A quote reflects the participants’ similar experiences:

*“Reflecting back, I think mostly about the fantastic meetings with all the people. It was in a way a bubble in time. That summer week was so special, it was as if life paused for a while, and there was so much love and beautiful friendship in the group, in combination with all the beautiful places and the fabulous weather — kind of a week in paradise …”* (IP15)

#### Subtheme: A Sense of Finding a new Family

Responding to a question on what she liked most about the programme, a participant responded:

*“What I carried with me from the first week, was the feeling of having gained a new family, that’s what it boils down to. Having a family of people that I previously did not know, and that I felt so comfortable and safe with these people is really kind of peculiar … I think that in many of the activities we did, step by step you get very close to each other. I did not think much about it while being there, but it felt like that was an underlying intention with the things we did, to support the development of trust between each other*.*”* (IP16)

Another participant was asked whether she felt respected by the others during the programme:

*“Yes 100%, I felt … I felt more seen there than by my own family, that’s how it is … (emotional) that’s how I feel*.*”* (IP10)

#### Subtheme: Nature, Programme Activities and Similar Life Stories as Catalysts for Deep Connection

Below, a participant reflects on how connection and affinity could develop so quickly in the group:

*“I think that you really have a direct understanding. When you tell something, then people understand it because they have been through similar things. And I think that by participating in something like this people become humble. I really felt that these were people with good hearts and with their feet firmly on the ground. In a way, people were very different, but we managed to fully meet each other here … and the adventure was what really made us so close, something that would have been different if we had done something else*.*”* (IP15).

A participant who had recently been treated for cancer expressed amazement that it was possible to connect with others so quickly.

*“Already the first evening, I took a short stroll along the beach with two other participants, we did not want to go to sleep and had a really nice talk … I had only known these people for a couple of hours, but we had deep conversations about relationships and cancer, which was very calming and comforting.”* (IP6)

For some participants, it was more challenging to connect to the larger group.

A participant who needed to take an alternative trail route on some days during the expeditions, due to visualvisual impairment caused by his cancer, expressed that he felt lonely, as the rest of the group went ahead of him. Asked about how he dealt with it, he said:

*“By expressing my feelings about it to the others, that I felt lonely and outside, it changed, and I became more included*.*”* (IP11)

A similar story was shared by another participant who had restricted mobility due to pain:

*“It was a bit hard for me and another person, one of whom I got on well with, while the other ‘group’ was very able and self-sufficient, and it did not feel so inviting for us to be with them …”* (IP3)

A year after the wilderness programme, most participants describe that they are still in contact with other participants. Mostly through social media, but also in person, as they travel to meet each other. One of the more socially engaged and active participants described this as follows:

*“I have contact with Henrik, met him a month ago or so. I was in Stockholm, and he came there too. So, I have contact with Ingegerd and Karin and Lisbeth, and also Kristina to some extent. Henrik has also been in my area a couple of times and then we have met when the time has been suitable.”* (IP14)

Participants came from all over Sweden, and the great distances between them were described as a challenge to maintaining contact.

#### Subtheme: Finding Personal Support in the Shared Stories

The context of programme participation in combination with meeting others with similar experiences also enabled deeper talks between participants. Listening to another participant’s story of how cancer had affected her, a participant understood that she was not alone in her mental struggles:

*“In a special talk that I had with Lottie and Carl, Lottie* *shares that she feels as if she does not have the right to be sad because there are people in even worse situations. I realised that others also felt that way, even though they had more serious cancer than me. My feelings have held me back from processing my own grief, so that was a very important conversation that I carry with me.”* (IP15)

Similarly, the topic of loneliness emerged at an early stage of the programme through group talks.

*“During the check-in and check-out, you had the opportunity to talk about your own situation and share your emotions. It was after about two days that we realised that all of us shared the feeling of loneliness in our everyday lives and that we felt that very few could relate to what we felt when talking about cancer, the traumas that we had experienced, or were experiencing, and here we had a group of people who actually could relate to me, so it was really good for me to meet a group of people who actually understand what I am going through.”* (IP17)

### THEME: Developing an Experience of Being Able and Competent in Nature

This theme relates to how participants share that programme participation supported their experience of becoming more able and competent in nature. It relates to both their physical and mental capabilities, but also to their growth as persons when they realise that by challenging themselves, they can succeed in doing what was previously perceived as almost impossible.

At a deeper level, a participant described programme participation as a life-changing event:

*“There is a before and after in my life … It feels as if my perspective has developed in many areas. I have gained tools that I really can use, both practically as well as emotionally. Being in the programme has in a sense given back a missing part in my life.”* (IP16)

#### Subtheme: An Experience of Developing Trust in One’s own Physical and Mental Capabilities

Being physically active was an essential part of the programme. At the beginning of the programme, participants expressed uncertainty about being able, as they were not aware of their own capacity. Their uncertainty was described as related to recent illness and treatment for their cancer or other conditions. The uncertainty and questioning of their own inability were described as progressing into certainty and a sense of being able and competent with each passing day of programme experience.

Being able to engage in physical activity was extra challenging for some participants. A participant described how general health problems (late effects of chemotherapy) had prevented him from being physically active for a long time, and that:

*“To me, it (hiking) was really hard because I have problems with my foot, and I am not very fit either, so it was very tiring, but on a personal level it was really rewarding to feel that I could manage this, more as the feeling of being able to trust myself.”* (IP17)

During the wilderness expedition, participants were coached to make choices that they found themselves comfortable with. For example, they could choose whether to carry a fully packed backpack along the hikes, or to travel lighter, to be more certain of their capacity.

*“I found it really good that they said we could go as light as possible, or as heavy as you wanted. You did not need to carry all your equipment, there were other options. It was in the way that you did not need to push yourself too much, but you could make your own choices. Some did feel: ‘that we will try it, we are testing this trail’, and it went well, and then they tried the second too, and it went well, and then you could stretch your limits a bit.”* (IP8)

Realising that it was possible to be able to manage physical activity was described in follow-up talks as a basis for trust in one’s physical and mental capacity. As a participant described in the follow-up talk:

*“With these positive memories that I actually can hike a bit, I can walk in rough, uneven terrain, I feel good thinking back on my experiences of being outside. Altogether it becomes a sprouting seed, that I may eventually be able to make my solo trip up north, after taking many small steps.”* (IP17)

#### Subtheme: Growing by Challenging One’s own Personal Boundaries in a Safe Context

Although “being challenged” was an underlying part of the programme, it became apparent that the concept of “challenge” differed between individuals. Often, but not always, their challenges were connected to consequences of their cancer experience. Having cancer and basically never been separated from family meant that it was a big challenge to be away from them for an extended time while having limited opportunities for contact. For others, with cognitive problems and a dependency on everyday routines, it was a challenge to let go of control and dare to sleep in a tent far out in the forest; and for some, this meant facing long-term fears, such as a fear of heights while climbing, or a fear of water while swimming or kayaking in open water.

During the wilderness expedition, participants were offered the opportunity to climb a fixed trail (clipped-in safely in a harness) that took them to an elevation about 20 metres higher, alongside a gorge. Encouraged to describe the climbing experience, 1 of the participants said the following:

*“One day while on the island we climbed. I am afraid of heights, but I did it anyway because it was kind of an easy trail that I felt worked for me, and with good support from Mike* *(facilitator) I made it. It was fun to dare!”* (IP15)

Encouraged to talk more about how and why she decided to climb, she adds:

*“First, I doubted if I would dare, should I really do this or not? Then I think he said something like, ‘this is how we can do it, and we can always abort if you cannot continue’. He was at my side while I climbed and explained where I should go, guiding me to feel safe.”* (IP15)

Being in and on the water presented a challenge for several of the participants, but it was also described as an opportunity for a structured personal challenge under safe, supervised conditions:

*“A substantial thing that became a kind of a theme for me was my fear of water, which had not been so obvious to me previously … It started on one of the first days, and I got some help from Kim and Leni (facilitators), and some of the fellow participants that had been swimming right before me. I was getting into the water, and I froze and panicked. I started crying, and I rarely cry in public, but being able to cry so early on the journey also helped me to feel supported in my fears. I think it helped me to dare more and more.”* (IP16)

After this incident, she was able to swim almost every day of the trip, and later in follow-up talks she also described how she had managed to go swimming in lakes together with her daughter, something she had promised her, but had never been able to do before. She described how the fear of water was connected to having cancer in her early teens and losing her hair because of chemotherapy.

Later in the expedition, during an instruction day to prepare for kayaking, at her own initiative (with the promise to her daughter in mind), she asked for support to make an assisted capsize with the kayak:

*“Here, I had this opportunity to practice this, so that I can play with her later. Mike (facilitator) helped me to tip over, and it was a terrible, panicking experience! It was so shallow that I could stand up, and it only took three seconds, but when I came up I started crying, most likely from both gratitude and relief, but I was not afraid of being in the kayak, only in the water.”* (IP16)

Another participant also described the sense of being challenged, due to being afraid while kayaking. She had never gone kayaking before and expressed being tense and having a strong fear of capsizing, but also a strong will to try to learn. Eventually, she did capsize, but came out of it stronger, and she could manage with the support of facilitators. After the first summer expedition, she developed an interest in kayaking and started kayaking back home with the local kayaking club.

#### Subtheme: A Growing Interest in wanting to be Active in Nature

Throughout the interviews, participants described how being part of the programme had given them the tools and skills to be out in nature by themselves. By learning more about camping chores, how to cook food, setting up a tent, and hiking in rain and sunshine, they describe having obtained skills and confidence to be able to manage. Through having tasted the outdoor life, participants expressed a growing interest in being outside. Being more physically active in nature might positively affect their quality of life in the longer term.

*“It made me more interested in nature, I would say, since in everything we did there are things that I definitively will do. Previously, I sensed that I would not have done it, but now I feel a lot more confident than before. Now I understand that it is not as hard as you may think it is, and that makes it easier.”* (IP2)

Yet another participant described how her programme participation supported her in becoming more active in a forest close to home:

*“I have a trail circuit where I walk several laps. It is close (to home) in case I get pain in my hip. I have recently learned that I am too flexible, and that stronger muscles stabilise the joint … Hiking poles supported me in the beginning, and now I manage without them.”* (IP9)

Some participants reflected on their motives for hiking out in nature and referred to a changed perspective. Through programme participation 1 participant learned that:

*“The journey is part of the goal. It’s important to stop and take pauses, and if we don’t get as far as planned, it’s not a failure. I have learned to stress down (relax) thanks to the programme, and can relax and take it easier, also while hiking, and not only after reaching the goal.” (IP8)*

A small group that became close friends during the expedition was inspired by the climbing element, choosing to engage in their own “climbing expedition” before coming to the follow-up basecamp event:

*“Well, before we came back to the second programme event, we went to Skuleberget in Docksta and did a long climb on the Via Ferrata … We would never have done that unless we had tried it earlier in the programme.”* (IP7)

### THEME: Nature, a Space that Supports Relaxation and Respite from Everyday Challenges and Stimuli

An apparent theme that was identified from the participants’ stories is that programme participation supported them in experiencing and realising that being in nature promotes relaxation and recovery from stress.

#### Subtheme: Peace and Calmness as Induced by Being in Nature

Asked about how nature affected them, participants expressed similar experiences:

*“You became very calm, very relaxed and content, genuinely happy, really happy in another way.”* (IP2)

*“It was so good being in the forest and having no contact with the outside world, so that’s something I’m bringing with me. It’s been so nice to just be able to relax.”* (IP9)

The same participant suffered on a daily basis from severe mental tiredness (cancer-related fatigue) and was asked whether this was also a problem during the expedition:

*“Well, a bit, but not as extensive as in the city with all these stimuli and so on, but you have stimuli there too (in the wilderness). But it mostly concerns sounds, all this sound you don’t really pay attention to, such as cars, yeah sound in general … Nature was calm and I felt so euphoric afterwards.”* (IP9)

#### Subtheme: Reconnecting to Nature’s Calming Properties after Programme Participation

Participants described how, when back home again, they more often choose to take walks in the close surrounding areas, both for the activity itself, but also to actively choose a path with more natural surroundings (forest, parks), to be able to experience silence, greenery and pleasant fragrances. Some had also been on longer hikes in national parks, bringing partners, family or friends along. Participants reflected on how hikes in nature affect their health. Below is a quote from a participant who struggled with their mental health, often triggered by fear of recurring cancer.

*“It has substantial positive effects on me, because it is when I am in nature on longer hikes that I can find peace in my mind. I have lots of worrying thoughts and catastrophic thinking and more, and when you are hiking in nature, this is the only thing that supports me to become grounded.”* (IP15)

A similar reflection was shared by another participant who also struggled with cancer-related mental and physical health:

*“When I started to become more stressed again, I realised I was less in nature, and I felt in my body that I needed to get out. I need to be in nature, it’s good for me and I can manage more psychologically … in other words, I do better when I can be physically active in nature.”* (IP5)

Programme participation was described as contributing to a better sense of self:

*“I often think about what this journey has done, and I am so grateful that I could be a part of it because I have learned to know myself better.”* (IP9)

Encouraged to give more detail, the participant added:

*“I mean the issue of setting limits. It’s a work-related thing that I have struggled with for many years, but what nature does to me I cannot really tell. It’s probably about finding peace and stillness, and experiencing that being in nature is good for you, and perhaps that you need to seek it out more often in your life, because that peace is what you need to be fine … Coming back home was such a contrast, stress, stress, to do this or that — when I think back (on the programme), it was a form of sanctuary.”* (IP9)

### THEME: An Experience of Becoming Genuinely Connected with Nature

The programme was deliberately designed to expose participants to nature’s diversity, to support them in experiencing nature unaffected, but also highly affected by human beings. The settings were a mix of cleared woodland, deep old forests, rocky shorelines, low mountains, sandy beaches, meadows, islands and sea waterways connecting some of these.

#### Subtheme: Old Forests and Uninhabited Shorelines as Support for Reflection, Learning and Being in the Moment

The experiences of forest bathing and hiking silently through an old forest induced moments for reflection and learning, shared by most of the participants.

In a description of silent walking, a participant said:

*“… the trees were majestic, no one had touched them, and it was so impressive, that they could be just as they were … and I thought … why can’t you just be allowed to be in the present.”* (IP9)

Another participant expresses the experience of kayaking for the first time in more remote nature:

*“The kayak trip was pure magic, one of the best things I have ever done! And there we did the same thing as in the forest, where we amongst other things did silent kayaking, just to listen … It became a totally new experience. I had never done kayaking in that way before … You move almost without making any sounds, only sliding through the water, and as a bonus the sun was shining for a while and put the moment in a special light.”* (IP8)

#### Subtheme: A Deliberate Choice to be Present, while Being in Nature

Several participants described how their perspective of themselves in relation to nature had changed after being in the programme. In a practical sense, they described how they had acquired more knowledge of hiking and camping, but also that, on a deeper level, they felt the call of the wild, and could enjoy being in nature in a new way:

*“After being in the programme I am fully convinced in a way that I was not before. Well, I knew nature was good for you, but now I feel that I don’t just know, I have tried it. I have a feeling inside that I just long for the forest. I can long to visit my parents-in-law who live by the sea.”* (IP8)

She continued, sharing an understanding that she used to see exercising as a chore but, in the programme, she developed a desire for activities in nature:

*“When you are in the forest or nature, you get it all. Both exercise and fitness training for the body, but also for the brain. Longing is my best way of explaining it. We have kayaks now, and I can long to get out kayaking, as it feels so great.”* (IP8)

Yet another participant described how the programme had made her look at nature differently:

*“I really want to be in nature, hiking and camping. It was positive for my soul, and I felt better when in nature. It sounds silly, but a new world has opened.”* (IP10)

She also described how she used to listen to music with her headphones when she took walks outside, before participating in the programme.

*“Now it feels cozier to be without music and listen to the birds and everything.”* (IP10)

Similarly, 1 year after participation in the expedition, 1 participant expressed:

*“It was such a nice experience. When I’m walking in the forest, I think of it, I mean to listen, listen to the birds, the moving leaves, and that we have nature. It means a lot to me to have a forest trail close by to walk along. That’s where I usually hike. It’s so nice …* (IP9)

A deeper perspective on the relationship between humans and nature was expressed by a participant:

*“It was interesting to see the different aspects of nature, and what happens where an area has been cleared and so on. It was exciting in many ways, the nature ecosystem. When you start to think about it, what happens with the animals and plants when you come into these old untouched forests. You feel different in your body there too. It’s hard to explain, but it becomes more of a journey in yourself. The journeys through the cleared areas are only a stretch from a to b.”* (IP18)

## Discussion

In the present study, childhood and AYA cancer survivors experienced how connection with and support from others, trust and self-confidence, personal growth, relaxation and recovery from stress were positively affected by WAYA programme participation. These identified themes are an indication that the WAYA programme impacted their health, well-being and ability to cope with cancer-related challenges in a broad sense, and in line with the programme aims that were targeted towards positive change across the 6 dimensions of Positive Health (see Figure 1). Few other studies of qualitative design have investigated the impact of wilderness programmes on the health of childhood and AYA cancer survivors.^20^ Some of our findings are in line with a previously published qualitative study by Stevens et al.^27^ about a wilderness expedition with 11 adolescent cancer survivors in a remote area of Canada. Identified themes in this study were: developing connections; togetherness; rebuilding self-esteem; and creating memories, the first 3 being along the same lines as some of the themes that emerged in our study. Comparison of our findings with a recent scoping review of what is known about the effects of nature-based interventions for adult cancer survivors in general shows similar results, in that nature is seen as an important resource to cope with cancer and supports relaxation, self-reflection and well-being, and facilitates social interaction.^42^ An overarching theme in our findings is that the WAYA programme supports personal growth, in part by establishing a deeper connection with other participants and by being challenged in nature. To the best of our knowledge, our study is the first to report that a wilderness programme may impact the personal growth of childhood and AYA cancer survivors. This finding can be understood in the model of Hendee/Brown.^43^ In their conceptual model, personal growth is defined as a range of effects toward expanded fulfilment of one’s capabilities and potential, and can be achieved if a wilderness programme meets 4 requirements: (1). Participants being in a receptive mode; (2). The right degree of stress from programme activities and from contact with the environment; (3). A change of pace and reprieve from many cultural influences that allow for a chance of attunement to oneself and the natural world; and (4). Opportunities for metaphors that increase participants’ awareness of desirable qualities that can be applied back home. These requirements were met in the WAYA programme. With respect to the first requirement, participants in our programme are in transition from young adulthood to adulthood and suffer from late and long-term effects of cancer. They are therefore likely to be motivated and receptive to change.^44^ Regarding the second requirement, the programme provided challenges in nature such as backpacking, kayaking and rock climbing that were adapted to the physical and mental condition of each participant.^31^ This ensured that all participants were able to encounter experiences to a degree that was challenging, but manageable and safe. Since the programme took place in the wilderness setting of the High Coast area of Sweden, with little to no opportunities for interaction with the outside world, the third requirement was also met. Furthermore, the programme provided metaphors by actively using the natural environment in mindfulness-based exercises, in group dynamics during challenging activities that required communication, navigation and coaching among participants, and in daily group sessions where experiences were shared and reflected upon. For example, in the first days of the wilderness expedition 1 participant expressed that he felt left behind because he was not able to hike at the same pace as the rest of the group, due to his visual impairment. His experience provided a metaphor for feelings of loneliness. When the group reflected on this, they found that loneliness was a feeling that appeared to be common among participants and connected to their experiences with cancer and cancer treatment. Subsequently, this mutual understanding of feeling lonely supported deeper connection and friendship among the participants. Experiencing loneliness is relatively common among childhood and AYA cancer survivors, and also a risk factor for anxiety and suicidal ideation.^15^ Another theme that was identified in the present study; “Nature as a space that supports relaxation and respite from everyday challenges and stimuli”, can be explained on the basis of 2 theories: the Attention Restoration Theory from Kaplan,^45^ and the Stress Reduction Theory from Ulrich et al.^46^ The Restoration Theory from Kaplan describes how exposure to natural environments can restore our limited cognitive resources, such as control of attention, that can become depleted by all the stressors and highly demanding tasks that we encounter in everyday life.^45^ This may be of specific benefit for childhood and AYA cancer survivors, since cognitive fatigue and cognitive dysfunction are among the most prevalent and distressing long-term effects they are reported to suffer from.^31,47,48^ In their theory, Ulrich et al.^46^ propose that natural environments can rapidly evoke positive emotions and reduce negative thoughts and physiological arousal, thereby enhancing recovery from stress.

Another theory that addresses nature’s health promotion effects, which is gaining more attention in relation to sustainability and the well-being of our planet,^49,50^ is the theory of nature-connectedness.^51^ Nature-connectedness can be defined as the affective, cognitive and experiential relationship that individuals have with the natural world.^52^ In the present study, nature-connectedness emerged as separate themes reflecting the different experiences when participants felt genuinely connected with nature. This was also demonstrated in the quantitative evaluation of the programme, where nature-connectedness was significantly increased for participants in the wilderness programme, compared to participants in the holiday programme.^29^ Previous studies have shown that nature-connectedness is strongly associated with psychological well-being,^53^ in the sense that individuals who feel connected to nature tend to be more psychologically balanced than others.^51^ It is suggested that the feeling of connection with nature is particularly related to eudaemonic well-being, a concept of well-being that is related to interest and personal growth.^54,55^ Several studies have shown that nature-connectedness is strongly linked to a sense of purpose, personal growth and development.^49,53,56^ It is this intrinsic connection between nature and the health and well-being of humans that is the basis of the ecosophy theory of Naess^33^ and the foundation of the WAYA programme in this study (Figure 1). Nature-connectedness and its contribution to eudaemonic well-being may in turn support the resilience and quality of life of childhood and AYA cancer survivors.^34,49,57^

### Strengths and Limitations

One strength of this study is that data on participants’ experiences was collected at several time points, ie, four times over a 1-year study period. We were thereby able to capture rich and diverse data about the participants’ experiences in the WAYA programme, as well as their own outdoor practices back home. Another strength was that interviews were performed by an independent researcher who was not part of the programme intervention. Furthermore, multiple and rich data sources, from all 19 participants, provided good information power, demonstrating that the sample size was large enough for this type of qualitative design.^40^ Another strength of this study was the inclusion of a diverse group of childhood and AYA cancer survivors from all over Sweden and with a broad range of medical variables, such as cancer diagnosis and physical disabilities. It was not only the physically active and more fit persons that participated in this study, but also those with considerable visual, hearing and walking impairments as a result of their cancer or cancer-related treatment.^31^

Our study also has limitations, as the intervention was in the phase of pilot testing. As previously reported, the first group of ten participants experienced some programme start-up challenges with the high pace during the first days of the expedition. The hiking pace was therefore adjusted in the second expedition for 9 out of 19 participants. Another limitation is that the intervention was conducted between the third and fourth wave of the COVID-19 pandemic in Sweden (June-September 2021), and consequently, participants had not been able to engage socially for more than a year. Their ‘need and greed’ to meet and connect with others may thus have influenced their experiences in the programme, and thereby the results of this study. Inevitably, in qualitative data analysis, the interpretation of data and the identification of themes is influenced by the personal and professional experience of the researchers. The first and last authors were facilitators in the wilderness programme and established a close relationship with the participants. It was therefore important to bring different professional perspectives into the analysis process, from other facilitators and an independent qualitative researcher. In addition, preliminary study findings were presented and discussed with a group of participants, to enhance the trustworthiness of the analysis and results. It should also be noted that the results of this study are not generalisable to the adult cancer survivor population and are restricted to the experiences of a small group of childhood and AYA cancer survivors in Sweden. Concerning the qualitative data analysis method, we also acknowledge a deviation from our previously published study protocol. ^32^ Originally, it was intended to apply a thematic analysis that follows the tradition of phenomenological hermeneutics.^58^ However, although rich and diverse, the interviews and the collected data were not fully directed towards phenomenological hermeneutical analysis of participants’ lived experiences. The semi-structured interview guide applied in this study was also intended to investigate the acceptability and content of the wilderness programme among participants. The questions and answers related to more practical issues of the WAYA programme may have given less room for participants to reflect more on their lived experiences.

### Recommendations for Practice and Future Research

Very little is known about the engagement of childhood and AYA cancer survivors with outdoor activities after they have participated in a wilderness programme.^20^ The findings of the present study provide some insights into the outdoor practice of childhood and AYA cancer survivors, and which WAYA programme-related activities they had incorporated in their daily lives back home. It appeared from this study that the programme provided participants with the tools and outdoor skills needed for them to feel safe and confident when out in nature on their own. The programme also provided them with the basic outdoor equipment to be borrowed and taken home after the intervention, which may have facilitated their engagement in outdoor activities. Another essential factor in support of their engagement in outdoor activities programme participation may be that through the coaching follow-up talks, they were able to find the outdoor activities that they liked best and that were also accessible in their direct living environment. This study shows that, for some, this was a hike in a park or forest close to home. For others, it was kayaking or overnight camping with family and friends. The findings of the present study suggest that a wilderness experience may be a promising health promotion intervention for interested childhood and AYA cancer survivors, and further exploration of the impact of wilderness programme participation for this population is warranted. To gain more in-depth understanding of how childhood and AYA cancer survivors connect with nature, and how their nature-connectedness can sustainably support their health and coping with cancer-related long-term and late effects in everyday life, we recommend that further qualitative research of different designs is needed. One such design could be a mixed-method approach where quantitative data on health outcomes and nature connectedness are integrated and merged with qualitative outcomes and narratives of childhood and AYA cancer survivors.^32^ Another study design worth further exploration is that of the visual method photovoice. Photovoice is a qualitative methodological approach that allows study participants to visually record and reflect on their experiences, with the goal of promoting knowledge about issues that are important to them.^59^ With the example of the WAYA programme, the aim of such a photovoice study could be to enable participants to reflect on their connection with nature in the programme, and to gain insight into how nature connectedness affects their health and well-being. As a third essential step in the photovoice methodology, they would then present their insights and needs to a wider audience of health care professionals, public health actors, community services and other stakeholders, who may have the ability to positively impact their access to such wilderness programmes.

This study provides essential information to guide the identification and selection of appropriate quantitative health-related outcome measures for RCTs aiming to investigate the effectiveness of the WAYA programme for the health and well-being of childhood and AYA cancer survivors. For example, the themes of connection with others, self-confidence and personal growth that became apparent in the present study are reflected within the domains of the Psychological Well-being Scale.^34^ We recommend that future RCTs or mixed-method studies of wilderness programmes for childhood and AYA cancer survivors include primary outcome measures that are aimed at measuring changes in their psychological well-being. Furthermore, we consider it important to investigate the impact of the wilderness programme and the individual outdoor practices of young adult survivors in the longer term. Future studies with a follow-up period of as long as 10 years are thereby warranted.

### Conclusions

Childhood and AYA cancer survivors experienced that a wilderness programme such as the WAYA programme supported their connection with others, and the development of trust and self-confidence, personal growth, and relaxation and recovery from stress. The WAYA programme provided them with the tools and skills to feel connected and safe in nature, which may have supported their engagement in outdoor activities after participating in the programme and coping with long-term and late effects of cancer and cancer-related treatment. Future studies may focus on gaining a more in-depth understanding of how nature-connectedness may impact their health and well-being, both in the short and longer term.

## Supplemental Material

Supplemental Material - Supporting Personal Growth in Childhood, Adolescent and Young-Adult Cancer Survivors Through Challenges in Nature — A Qualitative Study of WAYA Wilderness Programme Participation Supplemental Material for Supporting Personal Growth in Childhood, Adolescent and Young-Adult Cancer Survivors Through Challenges in Nature — A Qualitative Study of WAYA Wilderness Programme Participation by Mats Jong, Trine Stub, and Miek C Jong in Global Advances in Integrative Medicine and Health

## Data Availability

Data from qualitative interviews and follow-up talks contains sensitive information and is available upon request from the corresponding author.
