# Supplementary material for: Supporting Personal Growth in Childhood, Adolescent and Young-Adult Cancer Survivors Through Challenges in Nature — A Qualitative Study of WAYA Wilderness Programme Participation
Source: Glob Adv Integr Med Health. 2024 Mar 8;13:27536130241238150. doi: 10.1177/27536130241238150 (PMC10924559; doi:10.1177/27536130241238150)
Supplement: Supplemental Material - Supporting Personal Growth in Childhood, Adolescent and Young-Adult Cancer Survivors Through Challenges in Nature — A Qualitative Study of WAYA Wilderness Programme Participation [file sj-pdf-1-gam-10.1177_27536130241238150.pdf]

COREQ (COnsolidated criteria for REporting Qualitative research) Checklist

A checklist of items that should be included in reports of qualitative research. You must report the page number in your manuscript where you consider each of the items listed in this checklist. If you have not included this information, either revise your manuscript accordingly before submitting or note N/A.

Topic	Item No.	Guide Questions/Description	Reported on Page No.
**Domain 1: Research team and reflexivity**			
Personal characteristics			
Interviewer/facilitator	1	Which author/s conducted the interview or focus group?	p5
Credentials	2	What were the researcher's credentials? E.g. PhD, MD	p1, 7
Occupation	3	What was their occupation at the time of the study?	p1, 7
Gender	4	Was the researcher male or female?	p7
Experience and training	5	What experience or training did the researcher have?	p7
Relationship with participants			
Relationship established	6	Was a relationship established prior to study commencement?	p7
Participant knowledge of the interviewer	7	What did the participants know about the researcher? e.g. personal goals, reasons for doing the research	p6-7
Interviewer characteristics	8	What characteristics were reported about the inter viewer/facilitator? e.g. Bias, assumptions, reasons and interests in the research topic	p7
**Domain 2: Study design**			
Theoretical framework			
Methodological orientation and Theory	9	What methodological orientation was stated to underpin the study? e.g. grounded theory, discourse analysis, ethnography, phenomenology, content analysis	p5-6
Participant selection			
Sampling	10	How were participants selected? e.g. purposive, convenience, consecutive, snowball	p5
Method of approach	11	How were participants approached? e.g. face-to-face, telephone, mail, email	p5
Sample size	12	How many participants were in the study?	p5
Non-participation	13	How many people refused to participate or dropped out? Reasons?	p5
Setting			
Setting of data collection	14	Where was the data collected? e.g. home, clinic, workplace	p5
Presence of non-participants	15	Was anyone else present besides the participants and researchers?	No other prese
Description of sample	16	What are the important characteristics of the sample? e.g. demographic data, date	p5
Data collection			
Interview guide	17	Were questions, prompts, guides provided by the authors? Was it pilot tested?	P5, App 2
Repeat interviews	18	Were repeat inter views carried out? If yes, how many?	No
Audio/visual recording	19	Did the research use audio or visual recording to collect the data?	Audio, p5
Field notes	20	Were field notes made during and/or after the inter view or focus group?	yes, p6
Duration	21	What was the duration of the inter views or focus group?	p16, Inf Pow
Data saturation	22	Was data saturation discussed?	P16
Transcripts returned	23	Were transcripts returned to participants for comment and/or	No

Topic	Item No.	Guide Questions/Description	Reported on Page No.
		correction?	
**Domain 3: analysis and findings**			
Data analysis			
Number of data coders	24	How many data coders coded the data?	Three p 6
Description of the coding tree	25	Did authors provide a description of the coding tree?	Yes, Suppl 3
Derivation of themes	26	Were themes identified in advance or derived from the data?	p6, inductive
Software	27	What software, if applicable, was used to manage the data?	p6
Participant checking	28	Did participants provide feedback on the findings?	Yes p16
Reporting			
Quotations presented	29	Were participant quotations presented to illustrate the themes/findings? Was each quotation identified? e.g. participant number	Yes. p7-14
Data and findings consistent	30	Was there consistency between the data presented and the findings?	Yes. p7-14
Clarity of major themes	31	Were major themes clearly presented in the findings?	Yes. p7-14Yes
Clarity of minor themes	32	Is there a description of diverse cases or discussion of minor themes?	Yes. p7-14

Developed from: Tong A, Sainsbury P, Craig J. Consolidated criteria for reporting qualitative research (COREQ): a 32-item checklist for interviews and focus groups. *International Journal for Quality in Health Care*. 2007. Volume 19, Number 6: pp. 349 – 357

Once you have completed this checklist, please save a copy and upload it as part of your submission. DO NOT include this checklist as part of the main manuscript document. It must be uploaded as a separate file.

The thematic interview guide for the WAYA wilderness program (Originally in Swedish)
The interviews will be performed with an open thematic structure where the participants in a very broad sense will be asked to talk about and describe their experiences of being part of the wilderness program, and how they think about it now three months after program participation
Additionally, they will be asked to in-depth reflect on the context of the wilderness program, other participants, activities, and facilitators (as well as other volunteers).
In interviewing from a thematic structure it provides possibilities for follow-up questions such as: what do you mean by that, please elaborate, how did you think, what did you experience, what did you think made you feel that way, etc
The thematic areas give the general intention of the interviews that are in line with the objective, but dependent on what the participants choose to bring forward, other directions may open up.

Areas of example – Supportive questions
General perceptions

- Please describe your general experience of taking part in the program
- Please describe the best things about the program
- Please describe what was less good, or perhaps what you consider need improvement

Experience with program activities

- Hiking
  - Easy/challenging/hard
- Camping
  - How was your sleep?
  - Setting camp for the day?
  - Cooking?
  - Clothing, shoes, wetness, dirt, hygiene, dryness, coldness, warmness?
- Please describe your experience of the different program activities

        - Easy, hard, challenging, terrifying, impossible?
    - Please describe why you choose to participate in the different activities
    - Please describe why you choose NOT to participate in the different activities (if that occurred)
    - Was some things too challenging?
    - What experiences/emotions did you have during the activities?
    - Which activity was most rewarding/exciting/fun?
    - Please describe the least rewarding or hard activity

Facilitators

- Please describe how you perceive the different facilitators and volunteers in the program
- In what way did you experience support from them?
- Room/tips for improvement regarding support?

The other participants

- Please describe in general terms your perceptions/feelings about the other participants
    - Able to connect?
    - Easy/hard/challenges?
- In what way did you feel connected – or not?
- In what way did they respect you – or not?
    - Did they listen?
    - Were you taken seriously?
    - Did you listen to the other?
    - Did you take others seriously?
- Positive experiences of socialization?
- Negative experiences of socialization?

Nature – the High Coast area

- Please describe your general experience of being out in nature
- How do you feel about the area where the program took place?
- Were there any particular places or areas where you felt more comfortable than others?
- During your hikes or spending time in nature during the program – did you ever feel scared or worried about "being there"?
    - If so, how did you deal with it?

Health aspects

- Do you feel or notice that there is something in relation to your health or wellbeing that has changed during or after program participation?

Risk/incidents

During program participation…

- Did you ever feel unsafe?
    - If so, why, and how did you deal with it?
- Did you ever get hurt or hurt yourself?
    - If so, why, and how was it dealt with?
- Did you have an incident that you did not disclose to the facilitators?
    - If so, what happened and why did you choose not to tell anyone?

Motive for participation

- Why did you choose to participate in the program?
- If you would have been allowed to choose, Would you have chosen to be in the other group+ (Holiday intervention)
    - If so, your reason, and motive?
- What were your expectations on program participation?
- Expectations met?

- Would you recommend others to sign up for this kind of program
  - If so, why?

Supplement 3 Appendix Coding tree

All ***meaning units*** connected to the objective of the study: *"to explore the impact of the WAYA wilderness program on the health of childhood and AYA cancer survivors",* was identified, and sorted into descriptive domains, categories, and subcategories.

**Domain**	**Category**	**Subcategory**
1. Increase physical activity/being outdoors	Physical movement	Hiking, backpacking
2. Increase self-confidence/internal control	Challenge/risk activities	Sea-kayaking, rock climbing
3. Support personal growth	(Nature) reflective practice:	Mindfulness, meditation, forest bathing, journaling
4. Support building relationships	Free time/leisure activities:	Singing, swim/bath, fishing, campfire, reading, taking pictures, phone, playing games
5. Provide joy, balance, safety within nature<br>6. Increase self-efficacy/ self-care	Experiential activities:	Camping, outdoor skills, mapping/compass/orienting, trail cooking, safety skills training, equipment planning, foraging, 'Allemansrätten'*, leave no trace
